# The Effect of Melatonin on The Developmental Potential and Implantation Rate of Mouse Embryos

**Published:** 2012-12-12

**Authors:** Zakieh Asgari, Fatemeh Ghasemian, Mina Ramezani, Mohammad Hadi Bahadori

**Affiliations:** 1. Department of Biology, Faculty of Science, Payam e Noor University, Tehran, Iran; 2. Department of Anatomy, Faculty of Medicine, Guilan University of Medical Sciences, Rasht, Iran; 3. Faculty of Biology, Kharazmi (Tarbiat Moallem) University, Tehran, Iran; 4. Department of Biology, Faculty of Science, Ashtiyan Azad University, Tehran, Iran; 5. Cellular and Molecular Research Center, Faculty of Medicine, Guilan University of Medical Sciences, Rasht, Iran

**Keywords:** Development, Implantation, Melatonin, Differential Staining, Cleavage

## Abstract

**Objective:**

Melatonin is a scavenger agent that has been used to promote *in vitro* embryo development. This study was designed to show the effects of melatonin on the quality and quantity rate of preimplantation mouse embryo development and pregnancy.

**Materials and Methods:**

In this experimental study, super ovulated, mated mice were killed by cervical dislocation to collect two-cell zygotes from the oviduct of pregnant 1 day NMRI mice. Zygotes were cultured to the hatching blastocyst stage and the numbers of embryos at different stages were recorded under an inverted microscope. The cleavage rates of two-cell zygotes were assayed until the blastocyst and hatching blastocyst stage in drops of T6 medium that contained either melatonin (1, 10, and 100×10^6^, 10 and 100×10^9^ M) or no melatonin. The cell numbers of blastocysts were determined by differential staining, implantation outcomes were studied, and development and pregnancy rate were compared by the Chi-square (development) and Fisher’s exact (pregnancy rate) tests.

**Results:**

The addition of 10 and 100 nM melatonin to the embryo culture media promoted the development of the two-cell stage embryos to blastocyst and hatching blastocysts (p<0.01) and caused a significant increase in total cell number (TCN), trophoectoderm (TE), and inner cell mass (ICM) of the blastocysts (p<0.01). A difference was observed in the percentage of transferred embryos that were successfully implanted between the control and treatment groups (p<0.05).

**Conclusion:**

The data indicate that 10 and 100 nM of melatonin positively impact mouse embryo cleavage rates, blastocyst TCN, and their implantation. Therefore, melatonin at low concentrations promotes an embryonic culture system in mice.

## Introduction

The *in vitro* production (IVP) of mouse embryos is an important method for improving reproductive technologies and genetics. However, the developmental potential of embryos produced by IVP is still low, and optimization of embryo culture media would increase the production of developmentally competent embryos (1). The proliferation of fertilized eggs in culture conditions is arrested at the two-cell stage where free radicals are involved in the *in vitro* developmental block of two-cell embryos (2). The imbalance between the production of free radicals and a biological system’s ability to readily detoxify the reactive intermediates or easily repair the resulting damage is known as oxidative stress (OS). Disturbances in this normal redox state can cause toxic effects through the production of peroxides and free radicals that damage all components of the cell, including proteins, lipids, and DNA (3). The effects of OS depend on the size of these changes, with a cell being able to overcome small perturbations and regain its original state. However, more severe OS can cause cell death through necrosis, while even moderate oxidation can trigger apoptosis (4). Free radicals at physiological concentrations are also known to play a role in intracellular signaling, as it is involved in the normal processes of cell proliferation, differentiation, and migration (5). Even in the reproductive tract, free radicals play a dual role and can modulate various reproductive functions or lead to pathologies. Free radicals must be scavenged by antioxidants in the body. One of the antioxidants implicated to protect the body from free radicals is a hormone named melatonin (6). Melatonin is secreted by the pineal gland in the brain (7) and plays an important role in regulating the neuroendocrine system. This hormone is one of the major role players in the regulation of the circadian sleep-wake cycle. It is normally released from the pineal gland during the night in respo

The effect of melatonin on the *in vitro* developmental quality and quantity of mouse embryos, successful rate of embryo transfer, and subsequent pregnancy is not clearly elucidated. To evaluate the possible effect of melatonin on embryonic cleavage, developmental potential, blastocyst quality, and sequential embryo transfer, we have cultured mouse two-cell embryos in a development medium supplemented with different doses of melatonin until the blastocyst stage after which differential staining and embryo transfer were performed.

## Materials and Methods

### Animals

In this experimental study, a total of 35 female mice were housed individually in an air-conditioned room under a 12 hour light: 12 hours dark cycle (6 am: 6 pm), fed a commercial diet, and given water ad libitum. NMRI mice, 6-8 weeks old, were super ovulated by i.p. injection of 5 IU pregnant mare serum gonadotropin (PMSG; Organon, Holland) followed 48 hours later by an intraperitoneal (i.p.) injection of 5 IU human chorionic gonadotropin (hCG; Sigma, Germany). They were paired overnight with males of proven fertility. Two-cell embryos were mechanically obtained from their oviducts and collected in T6 medium and dishes that had been pre-warmed in an incubator at 37℃.

All the animal experimentation in this study was approved by the Guilan University of Medical Sciences (GUMS) Animal Ethics Committee.

### Animals

Mouse embryos at the two-cell stage were collected mechanically from oviducts of mated animals 46 hours after hCG injection. Embryos were collected and then washed three times in T6 medium (Sigma, Germany) supplemented with 4 mg/ml bovine serum albumin (BSA, Sigma, Germany).

### Experimental group

To assess the effects of melatonin (Sigma, Germany) on *in vitro* embryo development, 10-15 embryos were cultured in 50 µl of the T6 medium that contained either 0, 10, and 100 nM, or 1, 10, and 100 µM of melatonin. The melatonin stock solution was prepared with an ethanol/T6 system as follows: 23.23 mg melatonin was first dissolved in 0.1 ml absolute ethanol and 9.9 ml of T6 medium which resulted in a 100 µM concentration, then serially diluted in T6 medium. In this manner, we prepared the 1, 10, and 100 µM and 10 and 100 nM melatonin stock solutions. The control group contained no melatonin, whereas the 0.1% ethanol media was the vehicle group. The stock solutions were stored refrigerated at 4℃ for no longer than two weeks.

Embryos were then incubated for 4 days later to record the number of four, eight-cell embryos and blastocysts respectively.

### Determination of cell numbers in embryos

To determine blastocyst cell numbers from each group, embryos were placed in drops supplemented with 1 µg/ml of propidium iodide (Sigma, Germany) at 37℃ for 20-50 seconds. There were approximately 20 embryos that were in the late blastocyst stage per group. This was followed by incubation in 5 µg/ml of bisbenzimide (Hoechst 33342, Sigma, Germany) in absolute ethanol, overnight at 4℃. The propidium iodide stained only the nucleus of non-viable cells without an intact plasma membrane, whereas bisbenzimide stained the nucleus of both viable and non-viable cells. Hence, the trophoectoderm (TE) will be stained by both propidium iodide and bisbenzimide, whilst the intact inner cell mass (ICM) will be stained only by bisbenzimide. Embryos were mounted on microscope slides with glycerol, a cover-slip was placed on the top of the embryos, and they were initially examined to evaluate the number of cells. Under fluorescence microscopy (excitation filter at 420 nm, barrier filter at 365 nm), the outer TE cells were identified by the pink fluorescence of propidium iodide, whereas the ICM cells were recognized by the blue fluorescence of bisbenzimide. The numbers of ICM and TE nuclei were counted under an inverted fluorescence microscope (IX71, Olympus, Japan).

### Blastocyst development following embryo transfer

To assess the ability of late blastocysts to implant and develop *in vivo*, embryos were transferred to recipient mice. Female mice (C57BL/6, Razi Institute, Iran) were mated with a vasectomized male (C57BL/6) to produce pseudopregnant mice as recipients for embryo transfer. To ensure that all fetuses in the pseudopregnant mice were derived from embryo transfer (NMRI mouse) and not fertilized by black color mouse, we examined the skin color day 18 post-transfer.

In the control group, six blastocysts were randomly assigned to each uterine horn following developmental assessment during the *in vitro* culture. A total of 24 embryos from the control group were transferred to 4 recipients. On day 18 of pregnancy, the percentage of implantations was assessed.

Eight blastocysts from the group treated with 100 nM of melatonin were randomly assigned to each uterine horn following developmental assessment during *in vitro* culture. A total of 32 embryos were transferred per treatment to 4 recipients (Table 1). On day 18 of pregnancy, the percentage of implantations was assessed. The pregnancy rate was assayed in the embryos treated with the best dose of melatonin (100 nM), as determined by the differential staining assay, and the control group. In this study, better quality embryos were from the 100 nM melatonin-treated group.

### Statistical analyses

The outcomes of the development rate were assessed using the chi-square test. Pregnancy rate and fetal weight were assessed with Fisher’s exact test. All statistical analyses were performed using the Statistical Package for the Social Sciences version 16.0 for Windows. Differences were analyzed in cleavage rate, blastocyst and hatching blastocyst development rate, quality of blastocyst, and implantation outcomes, with a significance level of 0.05

## Results

In this study, 651 embryos at the two-cell stage were randomly cultured in six experimental and control groups (Table 2). When treated with 10 and 100 nM of melatonin, the rates of cleavage significantly increased compared to the control group. Our results indicated that the percent of morula formation was significantly higher in groups treated with 10 nM (94.24%) and 100 nM (91.24%) melatonin compared to the control group (80.64%, p<0.01). According to the results, the percentage of two-cell block decreased in groups treated with 10 and 100 nM of melatonin. The rate of blastocyst development significantly increased in the 10 nM (86.56%, p<0.01) and 100 nM (91%; p<0.001) melatonin groups compared to the control group (73.11%, p<0.01).

There was a significant increase in hatching percentage of embryos that were cultured in medium treated with 10 nM (61.46%, p<0.001) and 100 nM (58.2%, p<0.01) melatonin compared to the control group (43.01%, p<0.01).

 Absolute ethanol was used to dilute melatonin (0.1% as the vehicle group) and had no detrimental effects on the cleavage and development rates, and quality of embryos (p>0.05).

**Table 1:** Effect of different doses of melatonin on mouse embryonic development in comparison to the control group


Groups	Number of recipient mice	Numberof embryos	Number of pregnancies (number of embryos)	Fetal weight (mg)

Control	4	24	2 (1 and 3)	565 ± 77
100 nM of melatonin	4	32	3 (5, 6 and 6*)	622 ± 66*


*; p<0.05 vs. control group.

**Table 2:** Effect of different doses of melatonin on mouse embryonic development in comparison to the control group


Experimental groups	Ethanol concentrations (%)	Doses of melatonin	Numbers of two-cell	Numbers of morula (%)	Numbers of blastocysts (%)	Numbers of hatching blastocysts (%)

Control	0	0	93	75 (80.65)	68 (73.11)	40 (43.01)
Vehicle	0.1	0	97	77 (79.38)	72 (74.22)	41 (42.26)
Group1	0.1	100 µM	96	78 (81.25)	73 (73.95)	45 (46.87)
Group2	0.1	10 µM	108	90 (83.33)	81 (75)	52 (48.14)
Group 3	0.1	1 µM	111	92 (82.88)	86 (77.47)	53 (47.74)
Group 4	0.1	100 nM	134	123 (91.7)^**^	116 (86.56)^**^	78 (58.2)^**^
Group 5	0.1	10 nM	109	103 (94.4)^**^	99 (90.82)^***^	67 (61.46)^***^


^**^; p<0.01 and ^***^; p<0.001 vs. the untreated control group.

### Differential blastocyst staining

**Fig 1:**
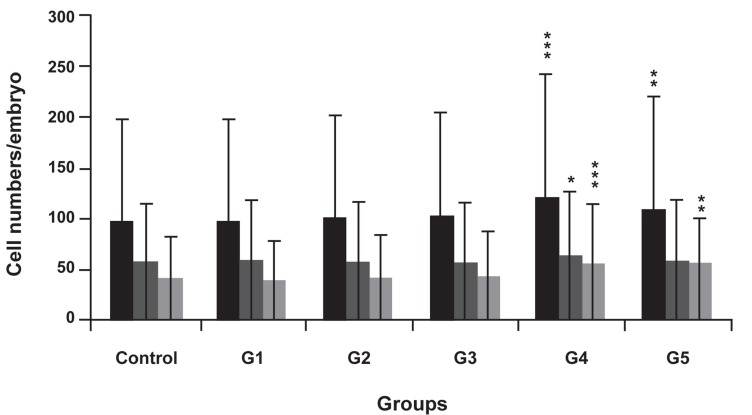
Effect of melatonin on embryonic cell number during IVP of two-cell embryos compared with the control group. Embryos were cultured in IVP medium that contained melatonin (G1: 100 µM, G2: 10 µM, G3: 1 µM, G4: 100 nM and G5: 10 nM). TCN in blastocysts, TE, ICM. ^*^; p<0.05, ^**^; p<0.01, and ^***^; p<0.001 vs. the untreated control group.

Blastocyst quality was promoted among the 10 and 100 nM melatonin-treated groups in comparison to untreated embryos (Fig 1). The total cell number (TCN), TE, and ICM of blastocysts treated with 10 and 100 nM of melatonin were higher compared to the control group (Fig 1). The mean TCN ± SD in the *in vitro* cultured blastocysts derived from two-cell embryos treated with 10 and 100 nM of melatonin were 109.6 ± 6.78 (p<0.01) and 121.4 ± 8.32 (p<0.001).The mean TE cells in these groups were 59.54 ± 5.98 and 61.64 ± 6.7 (p<0.05) and the TE cells were 50.06 ± 4.86 (p<0.01) and 59.76 ± 5.9 (p<0.001), respectively. The ICM: TCN percent was significantly higher in blastocysts treated with 100 nM (49.5%) compared to the control group (41.8%; p<0.01; Fig 1).

### Melatonin and pregnancy outcomes

Only embryos developed in the presence of 100 nM of melatonin were transferred to pseudopregnant recipients (Table 1). In the control group, there were two pregnancies, one that had one embryo and one with three. In the treatment groups there were 5, 6, and 6 embryos (these transfers were repeated 3 times for treatment group with 100 nM of melatonin). There was a difference between the control and treatment groups in the percentage of transferred embryos that were successfully implanted (p<0.05). The fetal weight was also higher in the treated group (622 ± 66 mg) than the control group (565 ± 77 mg; p<0.05).

## Discussion

We found that melatonin improved the development rate of mouse two-cell embryos when added at the two-cell stage. The rate of development to blastocyst was also significantly higher when embryos were cultured in T6 medium that contained melatonin. Ishizauka et al. (2) reported that the development rate of mouse embryos increased when embryos were cultured in BMOC-3 medium and melatonin was added 4 hours after insemination. In this study, embryos were cultured in T6 medium and melatonin was dissolved in ethanol. We added melatonin to the culture medium at the two-cell stage, when the embryos might have faced a twocell block. Furthermore, embryo development was assessed after *in vitro* culture of the two-cell embryos. The present data has demonstrated that melatonin decreased the two-cell block and increased the blastulation rate. Our results have shown the concentration dependent effects of melatonin on embryonic development *in vitro*. Thus, melatonin may be involved in metabolism at certain stages during embryogenesis to stimulate the formation of blastocysts. Reactive oxygen species (ROS) are involved in the two-cell block phenomenon in mice (2) and melatonin is an effective ROS scavenger (9).

Embryos face the risk of exposure to high levels of ROS during *in vitro* conditions. For example, oocyte aspiration, fertilization, and embryo culture could generate higher amounts of free radicals that negatively impact early embryonic development (1). To our knowledge, the present study is the first to report on the use of melatonin in the culture of mouse embryos at the two-cell stage using T6 culture medium. We have observed that melatonin promoted TCN, TE, and ICM at 10 and 100 nM concentrations, and also had a marked positive effect on cleavage and blastocyst rates. These results agreed with previous studies in that melatonin at a concentration of 10^-9^ M increased embryo development in bovines (10) and at 10^-6^ to 10^-8^ M in mice (2).

When the number of cells in the ICM of a blastocyst is decreased by approximately 30% or more, there is a high risk of fetal loss or developmental injury (11). The ICM cell number is also important for proper implantation, and thus a low ICM cell number may reduce embryonic viability (11). The TE cell also plays an important role in forming the placenta, and is required for mammalian cenceptus development (12). Reduction of TE cells causes embryonic viability and implantation suppression (13). Based on these observations, mouse blastocysts derived from two-cell embryos treated with 10 and 100 nM melatonin have resulted in increased TCN, TE, and ICM cell numbers. ICM and TCN also positively correlated with embryonic development during an embryo transfer assay (11). Our results showed that embryos treated with 100 nM of melatonin increased the implantation rate and embryo weight.

The researches of many scientists have been promoted culture systems of oocytes and embryos. For example, supplemented maturation medium with all-trans retinoic acid improved fertilization and development rates in a dose dependent manner (14). Culture in synthetic oviductal fluid promoted the potency of embryos to develop into blastocysts (15).

Physiological melatonin concentrations in the human blood are considered to be in the range of 100 pM to 1 nM (16), our results demonstrate that melatonin improves early embryonic development at physiological concentrations *in vitro*. In contrast, the presence of high melatonin concentrations (100 µM) have shown a decreased embryo development rate and inhibitory effect on the ICM/TCN ratio. Therefore, melatonin has a concentration-dependent effect on embryonic development, TCN, TE, ICM, and implantation rate.

## Conclusion

Results obtained in this study indicated that the addition of melatonin at concentrations of 10 and 100 nM promoted both the quality and the quantity of embryo development in the mouse culture system. In mice, a 100 nM supplementation of melatonin to the culture medium had a beneficial effect on the embryo development during developmental stages, TCN, TE, ICM, and implantation rate. Promotion and progression of embryonic development and the ICM/TCN ratio showed the concentration-dependent regulation pattern of melatonin in the mouse culture system.

## References

[1] Rodriguez-Osorio N, Kim IJ, Wang H, Kaya A, Memili E (2007). Melatonin increases cleavage rate of porcine preimplantation embryos in vitro. J Pineal Res.

[2] Ishizuka B, Kuribayashi Y, Murai K, Amemiya A, Itoh MT (2000). The effect of melatonin on in vitro fertilization and embryo development in mice. J Pineal Res.

[3] Henkel R, Maass G, Hajimohammad M, Menkveld R, Stalf T, Villegas J (2003). Urogenital inflammation: changes of leucocytes and ROS. Andrologia.

[4] Lysiak JJ, Zheng S, Woodson R, Turner TT (2007). Caspase-9-dependent pathway to murine germ cell apoptosis: mediation by oxidative stress, BAX, and caspase 2. Cell Tissue Res.

[5] Piantadosi CA (2008). Carbon monoxide, reactive oxygen signaling, and oxidative stress. Free Radic Biol Med.

[6] Berra B, Rizzo AM (2009). Melatonin: circadian rhythm regulator, chronobiotic, antioxidant and beyond. Clin Dermatol.

[7] Awad H, Halawa F, Mostafa T, Atta H (2006). Melatonin hormone profile in infertile males. Int J Androl.

[8] Du Plessis SS, Hagenaar K, Lampiao F (2010). The in vitro effects of melatonin on human sperm function and its scavenging activities on NO and ROS. Andrologia.

[9] Okatani Y, Watanabe K, Hayashi K, Wakatsuki A, Sagara Y (1997). Melatonin inhibits vasopastic action hydrogen peroxide in human umbilical artery. J Pineal Res.

[10] Poleszczuk O, Papis kwenta-Muchalska E (2005). An effect of melatonin on development of bovine embryos cultured in vitro under optimal or enhanced oxygen tensions. Reprod Fertil Dev.

[11] Shiao NH, Chan WH (2009). Injury effects of ginkgolide B on maturation of mouse oocytes, fertilization and fetal development in vitro and in vivo. Toxicol Lett.

[12] Craig JA, Zhu H, Dyce PW, Wen L, Li J (2005). Leptin enhances porcine preimplantation embryo development in vitro. Mol Cell Endocrinol.

[13] Kelly SM, Robaire B, Hales BF (1992). Pternal cyclophosphamide treatment causes postimplantation loss via inner cell mass-specific cell death. Teratology.

[14] Amiri I, Nasiri E, Mahmoudi R, Bahadori MH (2011). The effect of retinoic acid on in vitro maturation and fertilization rate of mouse germinal vesicle stage oocyte. Cell J.

[15] Hajian M, Hosseini SM, Asgari V, Ostadhosseini S, Forouzanfar M, Nasr-Esfahani MH (2011). Effec of culture system on developmental competence, cryosurvival and DNA fragmentation of in vitro bovine blastocysts. Int J Fertil Steril.

[16] Adriaens I, Jacquet P, Cortvrindt R, Janssen K, Smitz J (2006). Melatonin has does-dependent effects on folliculogenesis, oocyte maturation capacity and steroidogenesis. Toxicology.

